# Llangammarch Wells as a Health Resort

**Published:** 1896-10-03

**Authors:** 


### LLANGAMMARCH WELLS AS A HEALTH RESORT.

On the banks of the river Irvon in the county of Brecon stands the little village or hamlet of Llangammarch. It is literally cradled amid gently sloping hills, many of the hillsides being well-timbered and abounding in delightful walks. About half a mile from the village, and close to the edge of the river, is the famous barium spring, we believe the only one of its kind in this country. Like many similar spas, it was discovered by accident. Some half century ago, when the bed of the river was nearly dry, a labourer was hunting for a stray pig, and found it in a pool close by the barium spring, which was issuing from the bank below the usual water line. The labourer drank of the water, found it different from any he had ever tasted before, related his experience to some neighbours, with the result that investigations were made by and by. The waters soon obtained a local reputation in the treatment of various diseases, and, among others, chronic affections of the heart. Until within the last few years, however, the reputation of the waters does not seem to have spread much; but the statements of Bartholow, Flint, Böhm, and others that chloride of barium had a curative influence in certain diseases of the heart, and the wonderful results obtained at Nauheim under Schott, have brought the whole question of balneo-therapeutics very prominently before the public and the profession. Bartholow's success with chloride of barium in the treatment of aneurism would certainly warrant further trials at Llangammarch Wells and elsewhere.

The analysis of the water from the Llangammarch Springs shows it to contain chlorides of sodium, calcium, magnesium, lithium, ammonium, and, above all, barium, the latter being present to the extent of nearly six and a-half grains to the gallon. There are also traces of iron, potassium, strontium, and bromine. In addition to diseases of the heart, the Llangammarch treatment has been followed by much success in various gouty and rheumatic affections; and we should imagine it would be beneficial in most of those cases where there is, perhaps, no particular prominent disease, but where the patient is below par generally, and upset by business worries and prolonged city life.

We inspected the bathing arrangements, and are satisfied that the Schott treatment can be carried out as well at Llangammarch as at Nauheim, and it has the great advantage of being in our own island and more accessible. Moreover, Llangammarch is comparatively free from extremes of temperature, and a much longer "season" is possible there than at Nauheim. It might easily extend from March to November.

The hotel is within a hundred yards or so of the bath-houses and springs, and although small it is homelike and comfortable.

The medical department is under the charge of Dr. Black Jones, and the directors have been exceptionally fortunate in obtaining his services.

The sanitary arrangements are by Doulton, and the drinking water is taken direct from one of the mountain rills, conducted in pipes from the boundary of the estate.

Another point in favour of Llangammarch is its perfect quietness. There is an entire absence of tourists, German bands, and negro minstrels.

The visitor will find tennis lawns, golf links, two miles of a trout stream, a pond where rowing and canoeing can be indulged in, and there are several thousand acres of shooting, all reserved for those staying in the hotel.

The drawbacks to the place are the slowness of the train service and the small size of the hotel. A strong representation to the railway companies might do something for the former; and as to the latter, we understand that plans have been prepared for considerable additions and for the erection of a winter garden. It is to be hoped these will be ready for next season.

